# Tau PET imaging: present and future directions

**DOI:** 10.1186/s13024-017-0162-3

**Published:** 2017-02-20

**Authors:** Laure Saint-Aubert, Laetitia Lemoine, Konstantinos Chiotis, Antoine Leuzy, Elena Rodriguez-Vieitez, Agneta Nordberg

**Affiliations:** 1 Department NVS, Center for Alzheimer Research, Division of Translational Alzheimer Neurobiology, Karolinska Institutet, Novum 5th floor, 141 57 Huddinge, Sweden; 2 Department of Geriatric Medicine, Karolinska University Hospital Huddinge, Stockholm, Sweden

**Keywords:** Tau, Positron emission tomography imaging, Neurodegenerative diseases, Tracer development, Biomarker, Clinical research

## Abstract

Abnormal aggregation of tau in the brain is a major contributing factor in various neurodegenerative diseases. The role of tau phosphorylation in the pathophysiology of tauopathies remains unclear. Consequently, it is important to be able to accurately and specifically target tau deposits in vivo in the brains of patients. The advances of molecular imaging in the recent years have now led to the recent development of promising tau-specific tracers for positron emission tomography (PET), such as THK5317, THK5351, AV-1451, and PBB3. These tracers are now available for clinical assessment in patients with various tauopathies, including Alzheimer’s disease, as well as in healthy subjects. Exploring the patterns of tau deposition in vivo for different pathologies will allow discrimination between neurodegenerative diseases, including different tauopathies, and monitoring of disease progression. The variety and complexity of the different types of tau deposits in the different diseases, however, has resulted in quite a challenge for the development of tau PET tracers. Extensive work remains in order to fully characterize the binding properties of the tau PET tracers, and to assess their usefulness as an early biomarker of the underlying pathology. In this review, we summarize recent findings on the most promising tau PET tracers to date, discuss what has been learnt from these findings, and offer some suggestions for the next steps that need to be achieved in a near future.

## Background

The hyperphosphorylation and abnormal aggregation of tau, a microtubule-associated protein essential to neuronal stability and functioning, is implicated in various neurodegenerative diseases, labelled as *tauopathies*. The most common of these is Alzheimer’s disease (AD) [1]. One of the main pathological hallmarks of AD, along with the formation of amyloid-beta (Aβ) plaques, is the aggregation of tau into paired helical filaments (PHFs) and, subsequently, into neurofibrillary tangles (NFTs). Neuropathological studies have indicated that the regional distribution of NFTs follows a stereotypical pattern in AD, defined according to six successive “Braak stages” [2]: in the first two stages, NFTs are limited to the transentorhinal region (I-II), before spreading to limbic (III-IV), and isocortical association areas (V-VI). Other types of tau deposits are characteristic of various tauopathies. These deposits exhibit distinct regional distributions in the diseased brain [3], and may be composed of different tau isoforms. Indeed, there are six different isoforms of tau, formed by alternative mRNA splicing of the microtubule-associated protein tau (MAPT) gene. More importantly, the inclusion or exclusion of the exon 10 results in either 3 repeats (3R) or 4 repeats (4R) of the microtubule binding domain being transcribed in the tau protein, respectively [4]. While the 3R/4R ratio is 1:1 under physiological conditions and in patients with AD, tangle predominant senile dementia and chronic traumatic encephalopathy, 3R isoforms are dominant in Pick’s disease and 4R isoforms are dominant in corticobasal degeneration (CBD), progressive supranuclear palsy (PSP) and argyrophilic grain disease [5]. The role of tau aggregation in the pathophysiology of these neurodegenerative diseases, however, remains unclear. This is why the accurate, specific targeting of tau deposits in vivo in the brain would be highly valuable. However, this has historically been a formidable challenge for the scientific community.

**Fig. 1:**
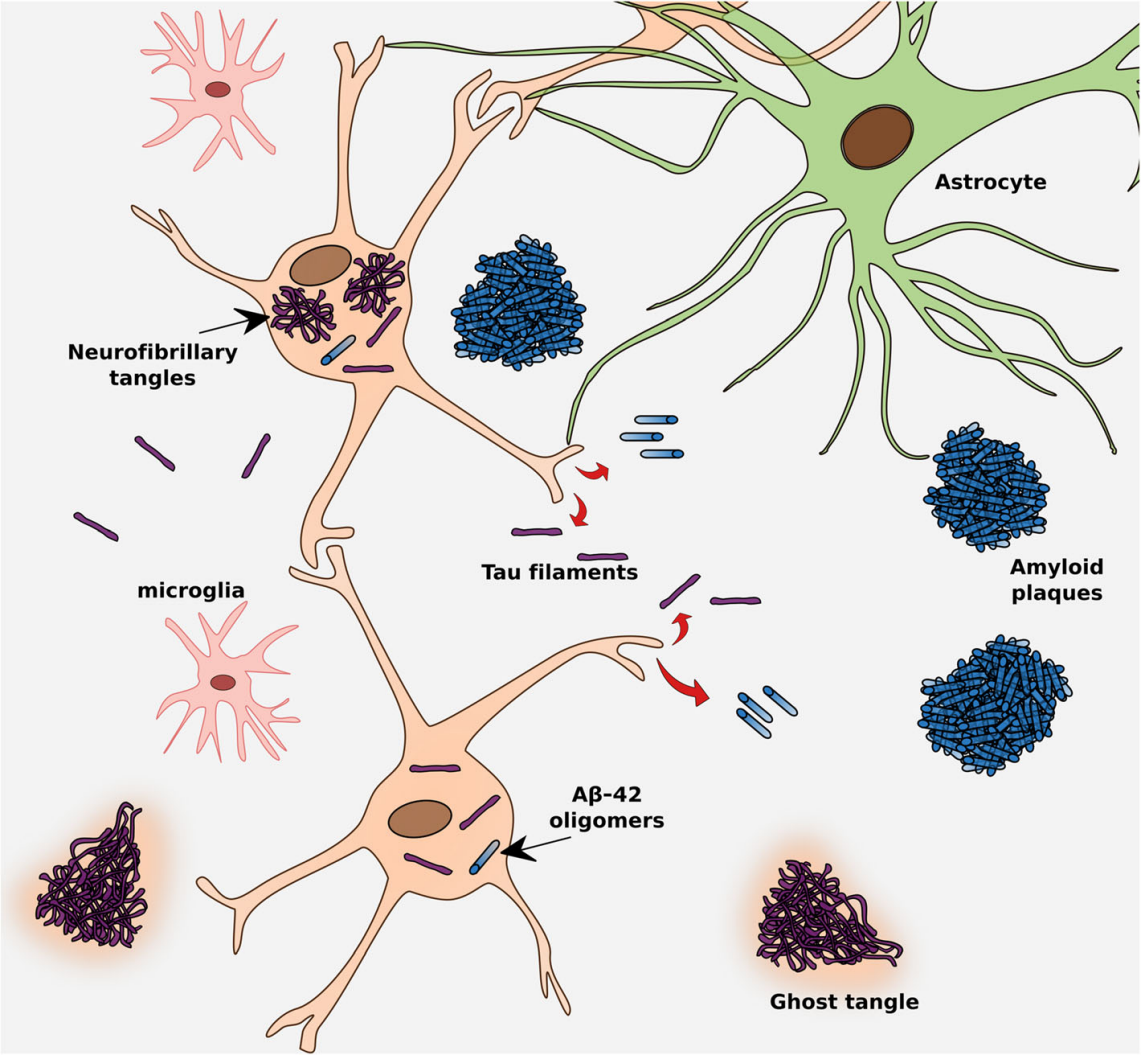
Tau pathology in relation to other pathological features in Alzheimer’s disease

Until very recently, it was only possible to observe tau deposits by immunohistochemistry in *post-mortem* tissue using specific antibodies, and the load of tau protein in the brain was only able to be measured in vivo using invasive indirect methods such as measuring the concentration of the protein (total- and phospho-tau) in the cerebrospinal fluid (CSF). Because tau – unlike Aβ plaques – aggregates primarily intracellularly (Fig. 1), it may be more difficult to access in vivo. However, over the past 5 years, a great effort has been ongoing to develop selective tau tracers for positron emission tomography (PET) imaging [6]. The emergence of promising tau-specific PET tracers, which are now available for clinical evaluation, has been a major breakthrough in research on AD and other related diseases. Specifically, it holds promise for exploring the regional patterns of tau deposition in vivo in different pathologies, discrimination between neurodegenerative diseases, and monitoring the spread of tau along disease progression. In addition, the combination of these tau tracers with other existing biomarkers bears great potential to help, in the times to come, discriminate between different pathologies, and, possibly, different tauopathies.

**Fig. 2:**
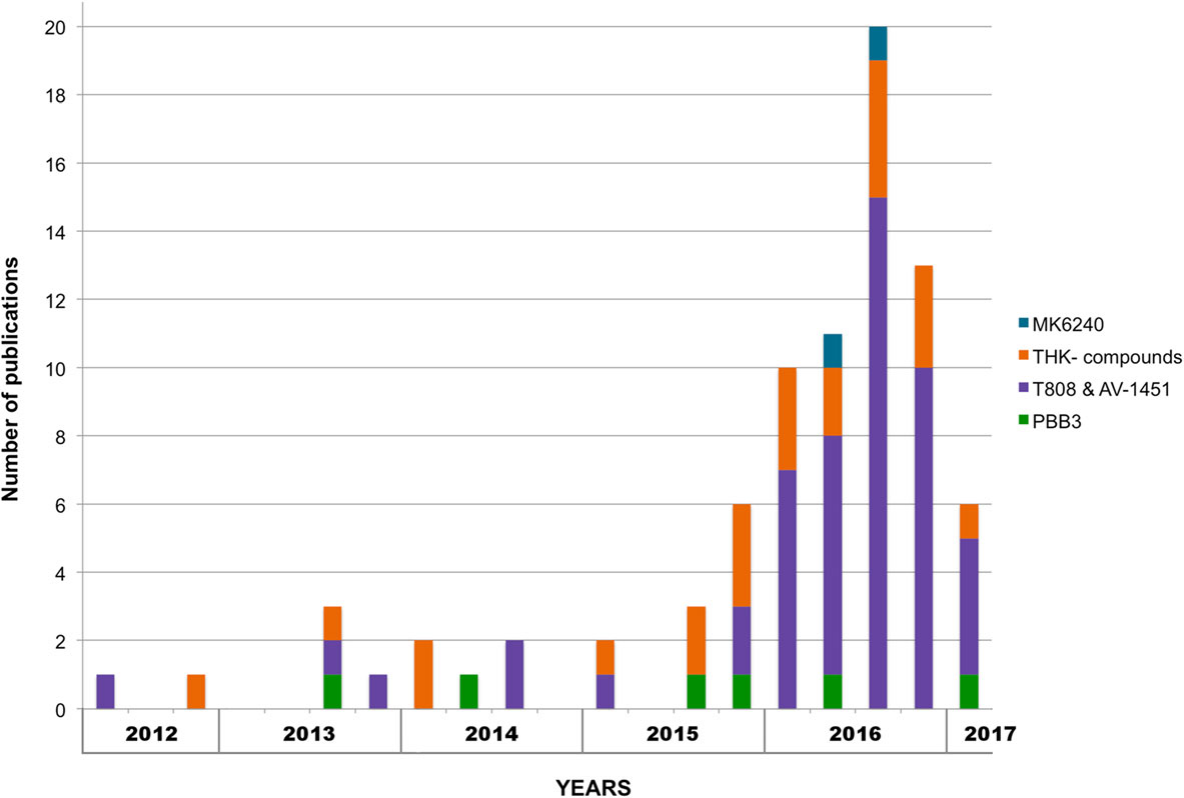
Number of publications on tau PET tracers in the recent years. The graph starts from the first publication on a tau tracer; each bar plot represents a period of three months

There is a fast increasing number of publications reporting findings on the recently developed tau PET tracers (Fig. 2). In this review, we summarize and discuss what has been learnt so far and suggest possible directions for the near future in tau imaging.

## Main text

### Development of tau-specific tracers

**Fig. 3:**
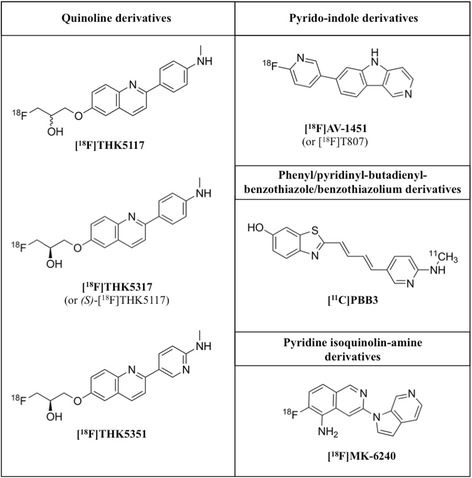
Chemical structures of the main tau-specific radiotracers. **[** ^**18**^ **F]THK5117**: 2-(4-methylaminophenyl)-6-[(3-[^18^ *F*]-fluoro-2-hydroxy)propoxy]quinoline; **[** ^**18**^ **F]THK5317**: *(S)-*2-(4-methylaminophenyl)-6-[(3-[^18^ *F*]-fluoro-2-hydroxy)propoxy]quinoline; **[** ^**18**^ **F]THK5351**: (*S*)-2-(4-methylaminopyridyl)-6-[(3-[^18^ *F*]-fluoro-2-hydroxy)propoxy]quinoline; **[** ^**18**^ **F]T808**: 2-(4-(2-[^18^ *F*]-fluoroethyl)piperidin-1-yl)benzo[4, 5]imidazo[1,2-*a*]pyrimidine; **[** ^**18**^ **F]AV-1451**: (7-(6- fluoropyridin-3-yl)-5H-pyrido[4,3-b]indole; **[** ^**11**^ **C]PBB3**: (5-((1*E*,3*E*)-4-(6-[^11^C]methylamino)pyridin-3-yl)buta-1,3-dien-1-yl)benzo[*d*]thiazol-6-ol; **[** ^**18**^ **F]MK-6240**: 6-([^18^ *F*]-fluoro)-3-(1*H*-pyrrolo[2,3-*c*]pyridin-1-yl)isoquinolin-5-amine

In addition to the characteristics required for a radiotracer to be a good candidate for a PET tracer [7–9], targeting cerebral tau represents an even greater challenge, for several reasons [10]. For example, tau is located both extra- and intra-cellularly, and hence the tracer must be able to cross the cell membrane. Over the past decade, several molecules have been suggested as potential tau PET tracers but many of those lacked sufficient specificity and selectivity [11, 12]. Based on both in vitro and in vivo results, three families of radiotracers have to date shown promise as specific tau PET tracers: the aryquinoline derivatives THK5117 (and the (*S*)-form THK5317) and THK5351, developed at Tohoku University, Japan [13–16]; the pyrido-indole derivative AV-1451 (also known as T807 and Flortaucipir), owned by Eli Lilly and originally developed by Siemens [17, 18]; and the phenyl/pyridinyl-butadienyl-benzothiazole/benzothiazolium derivative PBB3 (Chiba, Japan), derived from the same tracer family as the Aβ ligand Pittsburgh Compound B (PIB) [19, 20] (see Fig. 3 for chemical structures). This review focuses on these three families.

### Preclinical evaluation of the tau-specific tracers

#### Evaluation of tracers in vitro in brain tissue and *ex vivo* in animal model

**Fig. 4:**
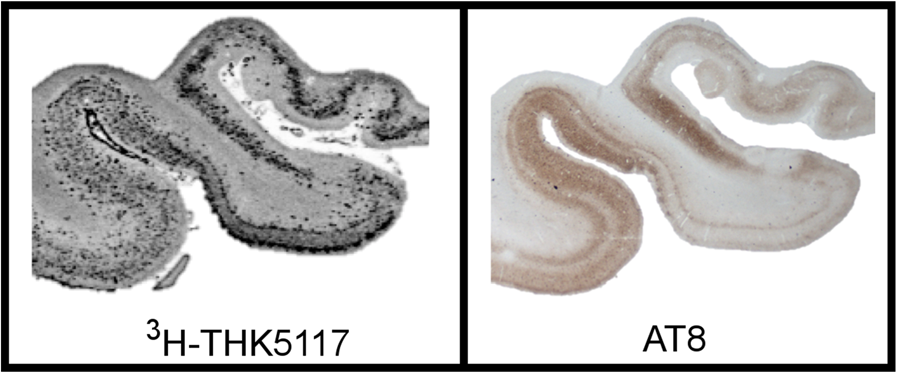
Comparison between [^3^H]THK5117 binding pattern using autoradiography and AT8 immunostaining. Experiments were performed on paraffin sections from the anterior part of the right hippocampus of a patient with pathologically confirmed AD. This figure was adapted from Lemoine et al., 2015 [24], with permission from the journal

The in vitro binding affinities of the promising tracers mentioned above have been well described in the literature, and the findings are summarized in Table 1. The binding affinity to tau deposits was determined using in vitro binding assays in AD brain homogenates and/or autoradiographies, depending on the tracer [14, 15, 21–24]. All tracers showed good affinity and exhibited a binding pattern on autoradiograms in human AD brain tissue, similar to the pattern of tau deposits revealed by immunostaining (Fig. 4) [15, 18, 24–28]. In addition, no selectivity towards Aβ was found when comparing to amyloid tracers [15, 18, 22–24, 26, 28–30]. A recent study reported that the binding pattern of [^18^F]AV-1451 corresponded better with the immunostaining pattern of some antibodies than others, suggesting that AV-1451 binds preferentially to mature tangles rather than pretangles or extracellular “ghost” tangles [31]. This illustrates that morphological differences may affect the binding intensity of tau tracers.

**Table 1:** Preclinical properties of the tau-specific PET tracers

		PBB3	THK5117			THK5351	AV-1451	
Radiotracer		^11^C	cold	^18^F	^3^H	^18^F	^18^F	^3^H
In vitro binding	Brain tissue	Kd_1_ = 2.5 [28] Bmax_1_ = 25nM [28] Kd_2_ = 100 [28] Bmax_2_ = 300nM [28]		Kd = 5.19 [15]; 11.5 [14] Bmax = 338 [15]	Kd_1_ = 2.2^a^; 3.1^b^ [24] Kd_2_ = 23.6^a^; 34.6^b^ [24] Bmax_1_ = 250^a^; 250^b^ [24] Bmax_2_ = 1416^a^; 1226^b^ [24]	Kd = 2.9 [26] Bmax = 368.3 [26]	15^f^ [22]	Kd = 1.4–3.72^c^; 0.63–1.70^d^ [23] Bmax = 15–62.5nM^c^; 46.9–119.7nM^d^ [23]
	Ki	Ki_1_ = 1.3^b^; 5.9^e^ [33] Ki_2_ = 23.5^b^ [33]	Ki_1_ = 0.001^a^; 0.0005^b^ [24] Ki_2_ = 27.4 [14]; 16^a^ [24]; 10.5 [15] Ki_3_ = 750^a^; 800^b^ [24]				Ki_1_ = 0.3^b^; 3.3^e^ [33] Ki_2_ = 97.2^b^ [33]	
Ex vivo biodistribution	LogP			2.32 [15]		1.5 [26]		1.67 [18]
	Uptake in mouse brain (2 min)			6.06%ID/g [15]		4.36%ID/g [26]	7.5%ID/g [29]	

^a^Tissue from hippocampal region; ^b^tissue from temporal region; ^c^tissue from frontal cortex; ^d^tissue from entorhinal cortex; ^e^tissue from motor cortex in a case of progressive supranuclear palsy; ^f^determined by autoradiography. *Aβ* beta-amyloid, *Bmax* receptor density, *ID* injected dose, *Kd* dissociation constant, *Ki* binding affinity, *LogP* partition coefficient. Ki and Kd values in nM and Bmax values in pmol/g (unless stated otherwise)

Furthermore, several derivatives have been developed, especially in the THK family, with the aim of improving their specificity. With the same goal, studies have compared the specificity of the enantiomeric forms of THK tracers. The results indicated that the *(S)*-forms had better pharmacokinetic and binding properties, as well as lower white matter binding than the corresponding *(R)*-forms, making the *(S)*-forms more suitable for in vivo investigations [14, 32].

In complement to in vitro investigations, ex vivo biodistribution and metabolite analyses have been performed for the three families of tau PET tracers. All the tracers showed rapid brain uptake and clearance through the liver, kidney and intestine [18, 20, 21]. However, in contrast to the other tracers, radioactive metabolites of [^11^C]PBB3 were found to enter the brain in a mouse model [20]. In addition, PBB3 is photoisomerized by fluorescent light, limiting the feasibility of in vitro experimentation and in vivo acquisitions with this tracer.

Direct comparison of tracers: There is a noticeable lack of head-to-head comparisons of the in vitro properties of tracers from different chemical families. To date, only one study compared the binding properties of [^18^F]AV-1451 and [^11^C]PBB3 [33]. Using brain tissue from several tauopathies, the authors reported partially distinct binding distribution of the tracers, as well as distinct selectivity for diverse types of tau deposits, with the binding of [^11^C]PBB3 to lesions comprised of 4-repeat or 3-repeat tau isoforms higher than that of [^18^F]AV-1451. In another study comparing the binding properties of [^3^H]AV-1451 and [^3^H]THK523 (an antecessor of THK5117 and THK5351) Cai et al. [34] reported that the two tracers showed a high affinity for distinct binding sites on the NFTs. Further investigations showed that those binding sites were different again from the thioflavine-T site targeted by [^3^H]PIB. Lastly, another in vitro study comparing [^18^F]T808 (a benzimidazo-pyrimidine derivative from the same family as AV-1451) with THK5105 (another antecessor of THK5117, and THK5351) suggested that the two tracers exhibited affinity to similar brain regions [29]. However, the binding properties of THK5117 and THK5351 have not yet been compared to AV-1451 and PBB3 binding in the same sample. Testing each of these tracers within the same cases would greatly improve the field’s knowledge regarding the affinity and specificity of tau tracers.

In vitro binding in different tauopathies: Studying the in vitro binding of available tau PET tracers in different non-AD tauopathies appears essential to our understanding of their specific targets in these diseases. This was investigated for PBB3 using autofluorescence binding studies, which showed specific binding to tau lesions in PSP, CBD and Pick’s disease, as confirmed by AT8 tau immunostaining [28, 33]. Similar results have been observed for THK5351 in both CBD and PSP [35, 36]. In contrast, mixed findings have been reported on the binding of AV-1451 in non-AD. While specific binding was reported to be absent or minimal in CBD and PSP, as well as in Lewy body-related cases [31, 33, 37, 38], and multiple system atrophy [31, 37], results for cases with Pick’s disease were conflicting: Marquié et al. [37] reported no specific binding of AV-1451, while Ono et al. [33] observed weak specific binding and Sander et al. [38] moderate specific binding in Pick’s disease brain tissue. The latter study also showed specific binding in two cases with mutations of the MAPT gene (FTDP-17) that exhibited 4R tau deposits, suggesting that specific binding may not be limited to 3R + 4R deposits. Both Lowe et al. [31] and Sander et al. [38] agreed, however, that specific binding in non-AD pathology is, whenever observed, less prevalent than in AD pathology. In addition, good correspondence was reported between visual assessment of AV-1451 binding and tau immunostaining [31, 37], although no significant correlation was found between AV-1451 binding and AT8 staining on quantitative assessment unless all tauopathies were combined in the analysis [38]. This further illustrates that not only morphological but also isoform differences could affect the binding intensity of tau tracers [31].

In vitro binding to non-pathological features: Marquié et al. [37] reported off-target binding of AV-1451 in neuromelanin-containing cells from the substantia nigra of PSP cases. This was confirmed by Lowe et al. [31], who more generally reported off-target binding in melanin-containing and vascular structures, as well as in the midbrain, meninges, scalp and basal ganglia in all cases, regardless of disease type. All structures reported to be specifically targeted by the tau tracers are summarized in Table 2.

**Table 2:** Targets of the tau tracers

	PBB3	THK5117	THK5351	AV-1451
Binds to	NFTs [28] (ghost tangles and non-ghost tangles [33]); neuropil threads [28, 33]; neuritic plaques [28, 33] and primitive plaques [33]; dense core amyloid plaques and diffuse amyloid-beta deposits [33]; Pick bodies [28]; astrocytic plaques [28]; tau inclusions in PiD, PSP and CBD [28]	PHFs tau [125]; NFTs[15, 24, 125](both intracellular and extracellular and ghost tangles [125]); neuritic plaques [125]; argyrophilic grains [125]; argyrophilic threads [125]; globose tangles [125]	NFTs [26]; thread-like structures in the white matter [35]; tufted astrocytes [36]	PHFs tau [18, 31, 37]; NFTs [23, 37] (both intracellular and extracellular [37], mature tangles [31] and ghost tangles [31, 33, 37]); neuritic plaques and primitive plaques [33] (to a limited extent); dense core amyloid plaques [33]; melanin-containing structures [31, 37]; lipofuscin-containing structures [31]; mineralized structures [31]; 3R + 4R tau deposits (much more than 3R or 4R [31]); MAO-A [23]
Does not bind to	Pretangles [33]	Pretangles [125]; alpha-synuclein lesions [125]; TDP-43 lesions [125]; Pick bodies [125]	Pretangles [125]	Pretangles [33]

*CBD* Corticobasal degeneration, *MAO-A* Monoamine oxidase A, *NFT* Neurofibrillary tangles, *PHF* Paired-helical filaments, *PiD* Pick’s disease, *PSP* Progressive Supranuclear Palsy, *R* Repeats (of the microtubule binding domain), *TDP-43* transactive-response DNA-binding protein 43

Overall, while all the tracers discussed here show good affinity for tau, the type of tau deposits (conformation, maturation stage, what tau isoform, etc.) and their specific binding site(s) are not yet fully known. The reported off-target binding may also represent a major limitation for interpretation of signal detection in vivo.

#### Preclinical in vivo characterization in animal models using micro-PET imaging

PET imaging in animals has been used to investigate the in vivo pharmacokinetic properties of newly developed tau PET tracers in wild-type mice, rats and monkeys, and in various transgenic mouse models expressing human tau.

Wild-type animal models: Cerebral retention of tau tracers in wild-type mice was investigated using micro-PET for all three families of tracers. All tracers showed rapid uptake and washout, indicating favorable pharmacokinetic properties [15, 18, 26, 28]. Because of its better binding properties and more rapid kinetics, the more recently developed [^18^F]THK5351 had a higher signal-to-background ratio than [^18^F]THK5117 [26]. Of note, a recent micro-PET study in wild-type mice investigated the effect of chirality on the kinetic properties of [^18^F]THK5105 (antecessor tracer). The authors showed that while both enantiomers had similarly fast initial uptake, the *(S)*-form had a more rapid washout, and therefore more favorable kinetics, than the *(R)*-form [32]. Interestingly, the difference between enantiomeric forms was more prominent in vivo than in corresponding ex vivo studies, possibly because of the effects of anesthesia on metabolism, according to the authors.

Steady accumulation of [^18^F]AV-1451 in bone was reported, probably due to defluorination of the tracer [18]. Possible off-target binding of [^18^F]AV-1451 was also investigated in the Rhesus monkey, using self-blocking (preinjection of a high dose of unlabeled AV-1451 prior to injection of [^18^F]AV-1451) [23]. Although this animal model did not exhibit tau pathology, the authors observed a significant decrease with time in the retention of [^18^F]AV-1451 throughout the brain, reflecting off-target binding of the tracer; further experiments suggested that this observation may have been due to binding to monoamine oxidase A (MAO-A). To further investigate in vivo the pharmacokinetic properties of these tracers in the presence of tau deposits, several studies have tracked the regional distribution and temporal evolution of tau pathology in small animal transgenic models expressing hyperphosphorylated tau.

Transgenic animal models: All of the discussed tau tracers have been investigated in distinct transgenic models. Micro-PET imaging was used to investigate the binding of [^11^C]PBB3 in the PS19 transgenic mouse model (expressing 4R tau pathology) [28], and the binding of [^18^F]THK5117 in two other mouse models with MAPT mutations: P301S (Tau-P301S) and biGT (bigenic GSK-3β x Tau-P301L) [39]. Both studies reported higher tracer uptake in transgenic mice than in wild-type mice, with in vivo retention significantly correlating with the corresponding in vitro patterns on autoradiography and AT8 immunostaining. In contrast, when [^18^F]AV-1451 retention was investigated using in vivo micro-PET in the APPSWE-Tau transgenic mice (carrying the human P301L tau mutation), cerebral retention was similar to that in wild-type mice [18], suggesting that these tracers do not bind to the tau aggregates present in these models.

One major drawback with these investigations is that different tracers were tested using different transgenic models, which precludes the comparison of the different studies and tracers. Additional limitations are inherent in the use of animal models *per se*. For instance, [^18^F]AV-1451 did not show significant retention when investigated in APPSWE-Tau mice [18]. Similar findings were reported for [^18^F]T808 in another transgenic mouse model of tau, also expressing P301L [29]. It was previously reported that the P301L mutation in transgenic mice only affects the 4R (and not the 3R) isoform of tau, and that the tau deposits in this model look structurally different from those in human AD pathology [40]. In addition, differences in post-translational modifications are likely to occur between mice and humans. The absence of [^18^F]AV-1451 binding in the APPSWE-Tau mouse model was thus probably due to its low affinity for 4R isoforms, and/or the mouse model not being suitable for investigating tau PET tracer binding in human tauopathies. Future in vitro studies investigating the type of tau deposits targeted by each tracer would greatly aid in the selection of mouse models appropriate for comparison of in vivo binding of the different tracers.

#### In vivo kinetic modeling in humans

In vivo kinetic modeling studies have been performed with tau PET tracers in humans, with the aim of determining an optimal method for quantifying tau retention. Because of the discernable interest in the clinical applicability of tau PET imaging, there is a need for quantification methods that can be easily transposed to the clinic. Though in vivo kinetic models using arterial sampling are the “gold standard” for accurate quantification of the pharmacokinetic properties of PET tracers, several studies have tested less-invasive quantification methods (i.e. without arterial sampling), using reference tissue models, more suited for use in clinical settings. In this respect, studies have also looked to validate semi-quantitative approaches such as the use of standardized uptake value ratio (SUVR), and to determine the optimal time interval for quantification.

Kinetic modeling in humans has been studied for all tracers (including the *(S)*-form of [^18^F]THK5117: [^18^F]THK5317, but not the racemic form) [41–46], and arterial sampling has also been used for all of them [43, 44, 46–48], except [^18^F]THK5351. These studies aimed at validating the optimum late-phase SUVR interval so as to quantify tracer retention. Cerebellar regions were selected as the reference tissue in all reference-tissue models because they are relatively spared from tau deposits in AD until late in the disease course [2]. Details of the findings are summarized in Table 3. An additional study reported the early-phase SUVR interval of [^18^F]THK5317 as a suitable proxy for brain perfusion [49].

**Table 3:** Summary of in vivo kinetic properties of tau tracers

Tracer	Publication	Population	Arterial sampling	Plasma-input kinetic model	Reference tissue models	Optimal time-interval for SUVR	Reference region
[^18^F]AV-1451	Wooten et al., 2016 [43]	4 CN, 3 TBI, 2 MCI due to AD	Yes	1TCM, **2TCM**, **plasma-input Logan,** **Multilinear Analysis 1**	**Reference Logan**	80–100 min	Cerebellum (excluding the vermis)
	Hahn et al., 2016 [47]	4 CN, 6 AD, 3 PSP, 2 CBS	Yes	1TCM, 2TCM, 3TCM, plasma-input Logan*	**SRTM2,** reference Logan*	80–100 min	Cerebellar cortex (excluding the vermis)
	Shcherbinin et al., 2016 [42]	4 young CN, 5 elderly CN, 5 MCI 5 AD dementia	No	-	reference Logan	80–100 min	Cerebellar crus
	Baker et al., 2016 [41]	5 young CN, 23 elderly CN, 15 AD dementia	No	-	SRTM, SRTM2, reference Logan	80–100 min	Cerebellar cortex
	Barret et al., 2016 [48]	4 young CN, 4 elderly CN, 8 AD dementia	Yes	1TCM, **2TCM,** **plasma-input Logan**	SRTM, **reference Logan**	80–100 min	Cerebellar cortex
[^18^F]THK5317 ((*S*)-[^18^F] THK5117)	Jonasson et al., 2016 [44]	4 MCI, 5 AD	Yes	1TCM, 2TCM*, **plasma-input Logan**	**SRTM,** reference Logan*	only interval tested: 70–90 min	Cerebellar cortex
	Betthauser et al., 2016 [70]	14 elderly individuals (ranging from CN to AD dementia)	No	-	SRTM, MRTM_2_*, reference Logan	30–50 min	Cerebellar cortex
[^18^F]THK-5351	Lockhart et al., 2016 [45]	6 CN, 10 AD dementia	No	-	SRTM, reference Logan	40–60 min	Cerebellar cortex
	Betthauser et al., 2016 [70]	24 elderly individuals (ranging from CN to AD dementia)	No	-	SRTM, MRTM_2_*, reference Logan	30–50 min	Cerebellar cortex
[^11^C]PBB3	Kimura et al., 2015 [46]	7 CN, 7 AD dementia	Yes	Single-input models, **Dual-input models (taking metabolites’ activity into account)**	**MRTM**_**0**_	30–50 min	Cerebellar cortex

All plasma-input and/or reference tissue models investigated in each study have been tabulated. Models that were described as “suitable” (i.e. for reference tissue models, those in which the reference-tissue model results agreed well with the plasma-input model results) are indicated in bold font. Plasmasinput and reference tissue models that were identified a best or most suitable are labelled with an asterisk (*). *AD* Alzheimer’s disease, *CBS* corticobasal syndrome, *CN* cognitively normal, *GM* grey matter, *MCI* mild cognitive impairment, *MRTM*_*0*,_ *MRTM*_*2*_ multilinear reference tissue models, *PSP* progressive supranuclear palsy, *SRTM* simplified reference tissue model, *SUVR* standard uptake value ratio, *TBI* traumatic brain injury, *TCM* tissue compartment model

The plasma-input Logan model was found to be suitable for determining retention of both [^18^F]AV-1451 and [^18^F]THK5317 [43, 44, 47]. The two-tissue compartment model (2TCM) was also reported as suitable in some studies [43, 44]. However, after testing different plasma-input compartment models, only dual-input models that took brain metabolite activity into account were found to be suitable for accurate quantification of [^11^C]PBB3 [46]. The reference-tissue model showing the best correlation with the output from plasma-input models for [^18^F]AV-1451 and [^18^F]THK5317 was the reference Logan model [44, 47]. For [^11^C]PBB3, despite the presence of radiolabelled metabolites capable of crossing the blood brain barrier, the multilinear reference tissue model (MRTMo) showed good correlation with the dual-input model [46]. Finally, different studies for every PET tracer investigated SUVR quantification. While there were some reservations about its use with [^18^F]AV-1451 because of nonlinear associations between SUVR values and reference-tissue model-derived parameters with this tracer [41], a recent study reported good correlation between SUVR over 80–100 min (the optimal time-window for all other studies) and plasma-input kinetic model-derived parameters [48].

Overall, the possibility of using, for all tracers, reference-tissue models and SUVR values as suitable measurements of in vivo binding is of great value for future applicability in clinical settings.

#### Other tau-specific tracers under preclinical evaluation

Additional tracers that appeared to be promising candidates for targeting tau deposits using PET include benzimidazole (lansoprazole and astemizole) [50], BF-126 or quinolone derivatives (BF-158 and BF-170) [51]. Recent in vitro experiments using the novel pyridine isoquinoline amine derivative MK-6240, released by Merck laboratories, have shown high affinity for NFTs, poor binding to Aβ plaques, and good grey matter/white matter binding ratios in autoradiography studies [23, 52]. Comparison with [^3^H]AV-1451 in autoradiography studies showed that [^3^H]MK-6240 provided greater contrast in binding between the hippocampus and subcortical regions and no off-target binding, and suggested that MK-6240 and AV-1451 might be competing for the same binding site. In vivo PET studies in the Rhesus monkey reported that [^18^F]MK-6240 displayed both rapid brain uptake and washout, indicating favorable tracer kinetics, and was also distributed homogeneously because of the negligible amount of tau in the Rhesus monkey brain [23, 52]. Further in vivo studies using self-blocking in the Rhesus monkey confirmed the absence of off-target binding in all brain regions for [^18^F]MK-6240, contrarily to [^18^F]AV-1451 [23]. Further investigation of these tracers in various tauopathies is needed, both in vivo and in vitro.

### In vivo assessment of tau using PET

#### In vivo assessment in cognitively normal individuals

In order to assess the specificity of tau radiotracers in vivo, PET studies have investigated their retention pattern in healthy subjects. Tau PET studies in cognitively normal (CN) elderly individuals using [^18^F]THK tracers have shown that cortical retention, although above reference levels, was relatively low and mainly confined to the medial aspect of the temporal lobe [45, 53]. Similar cortical findings were obtained using [^18^F]AV-1451, with all studies conducted thus far showing some degree of retention located within temporal regions [54–65].

In all these studies, however, locally high tracer retention was seen in a number of cerebral regions in CN subjects, both elderly and young, which seems to be off-target binding. For instance, studies have shown extensive in vivo binding of [^18^F]AV-1451 and [^18^F]THK tracers in the midbrain and basal ganglia, and of [^18^F]AV-1451, but not for [^18^F]THK5351, in the choroid plexus of CN subjects [26, 45, 53, 61]. As reported in in vitro studies (see above), this is likely to reflect off-target binding to various entities such as MAO-A [23], or pigmented or mineralizad vascular structures [31, 37]. In addition, high subcortical retention in the white matter was noted with [^18^F]THK5117, probably as a result of nonspecific binding to β-sheet structures present in myelin basic proteins [30]. This was greatly diminished, however, with the *(S)*-form of the tracer, [^18^F]THK5317, and with the more recently developed [^18^F]THK5351 [26, 53]. Lastly, high retention of [^11^C]PBB3 was reported in the dural venous sinuses of CN subjects [28]; it is not yet clear, however, whether this reflects off-target binding.

#### In vivo assessment in Alzheimer’s disease

Several clinical stages have been defined in AD, including preclinical, symptomatic pre-dementia (prodromal), and dementia. With the development of molecular imaging, specific diagnostic criteria integrating amyloid PET imaging have been recently proposed to better define these stages [66, 67]. It seems, however, that amyloid PET imaging alone does not discriminate well between symptomatic (prodromal and demented) stages of AD. There is thus a strong interest in investigating the regional retention of tau PET tracer in vivo at different stages of the pathology.

In patients with Alzheimer’s disease dementia: A fast growing number of in vivo studies aimed to assess the retention pattern of tau PET tracers in patients with a diagnosis of probable AD, in comparison to CN individuals. Most of the published studies in humans have thus far focused on the THK tracers or [^18^F]AV-1451; one study compared the in vivo retention of the radiotracer [^11^C]PBB3, however, in three patients with AD dementia and three CN subjects [28], reporting higher tracer accumulation in patients compared to controls in several brain areas, predominantly medial temporal regions.

The first THK radiotracers developed (the racemic forms of [^18^F]THK523, [^18^F]THK5105, and [^18^F]THK5117) showed important limitations, such as substantial overlap between clinical groups [68, 69] or high retention in white matter [30, 68, 69], which precluded simple visual assessment and prevented their future use in clinical settings. So far, the most promising radiotracers from this family appear to be [^18^F]THK5317 and [^18^F]THK5351. In vivo studies in AD dementia patients using these tracers have shown cortical uptake matching the distribution of tau deposits reported from histopathological studies, with retention in the inferior temporal region providing the best discrimination between patients and CN subjects [26, 53]. [^18^F]THK5351, however, has more favorable pharmacokinetics, less white matter binding, and a higher target-to-reference signal than [^18^F]THK5317 [70]. Other groups using [^18^F]AV-1451 in vivo substantiated these findings by reporting good discrimination between AD dementia patients and CN subjects, with greater cortical retention in patients, mostly within the temporal cortex [22, 45, 56, 59, 61]. The pattern of cortical retention in patients was again in agreement with the expected pattern of tau deposition in AD. Across studies, retention was predominant in the temporal cortex, with the inferior temporal gyrus appearing to be the best region for discriminating between AD dementia patients and CN subjects (Table 4).

**Table 4:** Cerebral regions showing significant group differences between AD patients and controls across studies

		CN (n)	AD (n)	Neocortex/Isocortical	Hippocampus	Parahippocampal gyrus	Inferior temporal gyrus	Superior temporal	Lateral occipital lobe	Posterior cingulate	Parietal cortex	Anterior cingulate	Putamen/striatum
[^18^F]T807	Johnson et al., 2016 [59]	56	6		NS	+	+			+			
	Cho et al., 2016 [57]	20	20	+	+	+	+	+	a	+	+	+	NS
[^18^F]THK5117	Harada et al., 2015 [30]	8	8	+	NS	+	+	+	NS	+	+	NS	NS
[^18^F]THK5317	data from Chiotis et al., 2016 [53]	9	9	+	NS	+	+	NS	+	+	+	NS	NS
[^18^F]THK5351	Lockhart et al., 2016 [45]	6	10		NS	NS	+		a	+	+	NS	NS

Only regions of interest that were comparable across studies are listed in this table

+ indicates that a significant difference between groups was reported; grey empty cells correspond to cerebral regions that were not reported in the publication

a: a significant difference was found when the whole occipital cortex was assessed. *AD* Alzheimer’s disease, *CN* cognitively normal, *NS* not significant

There is also an interest in the relationship between the patterns of tau deposition assessed in vivo and the symptomatology of clinical variants of sporadic AD, such as posterior cortical atrophy, logopenic variant of primary progressive aphasia, or behavioral/dysexecutive variant. Pathological studies have indicated that while these atypical forms share the pathological hallmarks of AD, they present with distinct neurodegenerative patterns, matching the symptomatology [71, 72]. Case series describing the retention of [^18^F]AV-1451 in vivo in posterior cortical atrophy, logopenic variant of primary progressive aphasia, and a behavioral variant of AD [61, 73, 74] as well as in one non-amnestic AD patient [61], have reported a neuroanatomical correspondence between the retention of the tracer and the clinical presentation for all variants, with [^18^F]AV-1451 retention most prominent in the clinically affected regions.

In prodromal Alzheimer’s disease and mild cognitive impairment: Beyond the ability to discriminate AD dementia patients from CN subjects, a major challenge for tau radiotracers is their efficacy as early biomarkers, that is, their use as a sensitive tool for detecting early stages of AD tau pathology. In a recent study using [^18^F]THK5317, the authors reported that not only patients with AD dementia but also prodromal AD patients (patients with mild cognitive impairment – MCI – and positive Aβ PET) had significantly greater cortical retention than CN subjects [53]. There was however no statistical difference between prodromal AD and AD dementia patients in this sample, although a greater proportion of patients with AD dementia showed high [^18^F]THK5317 retention in cerebral regions that are expected to be affected by tau pathology only late in the disease course. Other studies have reported that [^18^F]AV-1451 retention best discriminated MCI patients from CN subjects in mesial temporal regions (parahippocampal cortex, and entorhinal cortex) [56, 59]. As for the hippocampus, interestingly, some authors reported significant group differences [56] while others did not [59]. This discrepancy was probably due to differences between the studies in quantification methods and the studied populations: other than the differences in recruitment criteria, not all MCI patients in these two studies were amyloid positive (77 and 67%, respectively), meaning that a significant proportion were unlikely to be at an early stage of AD. In addition to these findings, Pontecorvo et al. [75] reported that younger AD patients (i.e. under 75) had greater [^18^F]AV-1451 cortical retention than older AD patients, and Cho et al. [57] reported that patients with early-onset AD (i.e. < 65 years) had greater [^18^F]AV-1451 cortical retention than patients with late-onset AD, as described in *post-mortem* histopathology studies on NFTs and neuritic plaques [76]. Of note, the same off-target binding reported in CN subjects was also observed in AD patients for all tracers [26, 28, 53, 61].

Relationship between the retention of the tracers and clinical impairment: Several studies using [^18^F]AV-1451 or THK radiotracers have started investigating the relationship between the regional tracer retention and concomitant cognitive performance in AD patients. They have reported a significant negative relationship between global cortical tracer retention and global cognitive status [56, 68], and also between retention in the temporal cortex and global cognition [30, 59, 77]. One longitudinal study also reported a significant positive relationship between increased [^18^F]THK5117 retention in the temporal cortex and cognitive decline [78]. Retention in the temporal cortex was also found to correlate with memory impairment in AD patients (across both prodromal and dementia stages) [57, 77]. Specifically, it appears that worse performance on domain-specific tests was associated with greater retention in key regions implicated in the involved cognitive domain [56, 61].

In preclinical Alzheimer’s disease: Conceptual and biomarker advances over the past decade have led to the identification of a preclinical phase of AD, recently formalized by new diagnostic criteria that integrate biomarkers for brain amyloidosis (i.e. CSF Aβ_42_ and Aβ PET) and neurodegeneration (CSF tau, regional atrophy, and [^18^F]fluorodeoxyglucose ([^18^F]FDG) PET) [66, 67, 79, 80]. Though these criteria for preclinical AD have not been formally applied in all studies that have thus far used tau PET imaging to investigate CN older adults, Aβ-negative subjects had only localized increases in medial temporal lobe retention, while Aβ-positive subjects, believed to be within the AD preclinical pathway, showed more extensive tracer retention, including in AD signature regions [54, 64]. Comparison between Aβ-positive and Aβ-negative subjects, however, showed no group differences in hippocampal retention [64]. A further study involving sub-classification of subjects into preclinical stage 1 (Aβ-positive, neurodegeneration-negative) and preclinical stage 2 (both Aβ- and neurodegeneration-positive) showed higher [^18^F]AV-1451 retention in medial temporal regions at both stages 1 and 2, relative to Aβ-negative and neurodegeneration-negative subjects (stage 0), and higher levels in the inferior temporal gyrus at stage 2, relative to stages 0–1 [60].

Of note, a highly interesting population to study preclinical stages of AD in is presymptomatic individuals carrying mutations involved in autosomal dominant AD. These individuals have been the focus of many research groups over the past years, as they will eventually develop AD, and thus offer the opportunity to assess in vivo the progression of pathological features before the onset of symptoms [81, 82]. There are to date, however, no published reports on tau PET in presymptomatic cases of autosomal dominant AD.

#### In vivo assessment in non-AD proteinopathies

CBD and PSP, two diseases in the spectrum of frontotemporal lobar degeneration, which are characterized by atypical parkinsonism and substantial clinicopathological overlap [83, 84], have received increased attention with the emergence of tau PET imaging. Both diseases are characterized by the deposition of abnormally hyperphosphorylated tau, mostly 4R, in tubular or straight filaments, in contrast to the PHFs in AD. Moreover, the spatial distribution of tau deposits in these diseases is distinct from that seen in AD [85, 86]. High tau deposition (measured with [^18^F]AV-1451, [^18^F]THK5317 or [^18^F]THK5351 PET) was observed in patients with a clinical diagnosis of PSP, in areas expected based on the neuropathological literature: the basal ganglia, thalamus, dentate nucleus of the cerebellum, and midbrain [36, 53, 87–89]. The association between [^18^F]AV-1451 retention in the basal ganglia and clinical deterioration in these PSP patients was not consistently reported. Concordance with pathological patterns of tau deposition was also found in patients with CBD: case-reports of Aβ-negative patients with clinical diagnoses in the CBD spectrum revealed increased tau deposition, as measured by [^11^C]PBB3, [^18^F]THK5317 and [^18^F]THK5351, predominantly in white matter and the basal ganglia, but also in other cortical areas [28, 35, 53].

Dementia with Lewy bodies and Parkinson’s disease are characterized by the presence of α-synuclein aggregates, although the presence of tau deposits similar to those in AD pathology are also commonly found [90–92]. [^18^F]AV-1451 retention in patients with dementia with Lewy bodies and Parkinson’s disease-related cognitive impairment, but not in cognitively unimpaired Parkinson’s disease patients, was found to be higher than in controls, although greatly variable [93]; the [^18^F]AV-1451 retention was negatively related to global cognitive function but not to the concomitant Aβ load. Another study comparing patients with dementia with Lewy bodies and patients with AD dementia reported a much lower cerebral retention of [^18^F]AV-1451 in AD, and revealed that the retention in the medial temporal lobe could discriminate between the two disease groups [94]. Though further studies are required, and while keeping in mind that the clinical distinction between dementia with Lewy bodies and AD can be challenging, these findings highlight the potential utility of tau imaging in the context of differential diagnosis.

Following a different approach, Hansen et al. and Cho et al. [88, 95] took advantage of the reported off-target binding of [^18^F]AV-1451 to neuromelanin [37], and aimed at imaging the loss of dopaminergic neurons in the substantia nigra of patients with Parkinson’s disease [88, 95]. Lower [^18^F]AV-1451 nigral retention was observed in patients with Parkinson’s disease, in comparison to a control group, although the overlap between patients and controls limits the clinical translation of the findings. Further, nigral retention in patients with Parkinson’s disease did not correlate with dopamine transporter levels in the basal ganglia (measured by [^123^I]FP-CIT single photon emission computed tomography), motor disability, age, or time since diagnosis.

In vivo retention of [^18^F]AV-1451 was also assessed in cases carrying mutations of the MAPT gene: Bevan-Jones et al. [96] described, in a patient with familial frontotemporal dementia due to a MAPT mutation (MAPT 10 + 16C > T), a retention pattern in agreement with the regional pattern of 4R tau pathology observed in the brain of the deceased father, carrier of the same mutation. Smith at al. [97] studied the in vivo retention of [^18^F]AV-1451 in three symptomatic patients (two with MCI, one demented) carrying a MAPT mutation (p. R406W); the latter mutation is pathologically characterized by the presence of cortical NFTs. Here again, the [^18^F]AV-1451 retention pattern was in agreement with reported post-mortem findings on tau deposits, showing involvement of temporal and frontal regions with sparing parietal and occipital lobes [98]. The authors suggested a progression pattern of tau in this mutation, although this requires further investigation in studies with a longitudinal design and larger sample sizes.

Taken together, these studies suggest that the developed tau PET tracers can image the expected regional distribution of tau pathology outside the AD spectrum, especially in tauopathies. This is, however, at odds with in vitro findings mentioned earlier, which suggests that [^18^F]AV-1451 might not bind substantially to, or might bind only to a small fraction of, the 4R tau burden [31, 37].

#### In vivo assessment in suspected non-AD pathophysiology

Operationalization of the National Institute on Aging-Alzheimer’s Association (NIA-AA) criteria for preclinical AD [79] led to the identification of Aβ-negative CN individuals with positive neuronal injury biomarkers [99]. Believed to represent non-AD etiologies, this group has been labeled “suspected non-AD pathophysiology” (SNAP). SNAP is thought to represent the in vivo equivalent of the recently described “primary age-related tauopathy” (PART), a concept currently under debate [100], introduced to describe the frequent observation in autopsy studies of focal NFTs pathology, despite the absence or minimal presence of Aβ plaques [101]. Several investigations using tau PET have made reference to SNAP as a possible explanation for the high percentage of Aβ-negative cases in CN individuals with an estimated Braak stage of I-II [56] and for focally elevated cortical [^18^F]AV-1451 retention [57, 63]. Additional studies have described cases possibly representative of PART [62, 95], although these also raised the possibility that AD pathology might be masking PART in preclinical individuals, with Aβ pathology below the detection threshold of Aβ PET imaging. Findings from the Harvard Aging Brain Study, however, do not support the hypothesis that SNAP is the in vivo counterpart of PART, as mean retention of [^18^F]AV-1451 within the medial temporal lobe among SNAP individuals was almost identical to that seen in stage 0 subjects (CN, Aβ- and neurodegeneration-negative) and lower than levels in subjects at preclinical stages 1–2 [60]. Importantly, this study highlights discordance between tau PET and neurodegenerative biomarkers used to define SNAP (i.e. hippocampal volume and [^18^F]FDG PET), a finding that carries implications for staging criteria for both SNAP and preclinical AD.

### Staging based on tau PET

To date, three cross-sectional studies have attempted to explore the spreading pattern of [^18^F]AV-1451 tau PET retention while translating the post-mortem staging system of tau pathology in AD from Braak and Braak [2] to in vivo staging models [54, 56, 62]. Secondary aims of these studies included exploring the relationship between these estimated in vivo Braak stages, other biomarkers (including amyloid PET and grey matter changes), and cognition.

All three studies included both CN and cognitively impaired individuals and employed either classification models or thresholds for classifying individuals with abnormal [^18^F]AV-1451 tau PET retention in selected regions of interest (ROIs). Measurement of the prevalence of abnormal [^18^F]AV-1451 retention (defined as [^18^F]AV-1451 positivity) in these ROIs indicated that the medial temporal lobe was the region most prominently affected across participants, followed by the adjacent temporal neocortex, the neocortical association areas and, the primary cortices [56], consistent with the stereotypical progression of tau pathology described by *post-mortem* studies [102]. Based on [^18^F]AV-1451 positivity in the selected ROIs, the authors assigned theoretical Braak stages to the participants. According to that staging, the majority of symptomatic individuals with a positive Aβ PET scan (Aβ-positive MCI or AD patients) were classified as Braak stage V-VI [54, 56, 62]. Surprisingly, however, a relatively large number of Aβ-positive MCI patients were classified as Braak stage 0 in one study [62]. Of note, not all patients could be staged in the theoretical models [56, 62], as also occurs with neuropathological evidence [2], and these were classified as “variants”; most variants were Aβ-positive [62]. Taking into account both CN and cognitively impaired individuals, the estimated Braak stage was associated with cognitive performance [56, 62]. Moreover, in a large group of young and elderly CN subjects, [^18^F]AV-1451 retention in ROIs created to match the neuropathological “Braak” stages was related to poorer cross-sectional memory and global cognitive performance, as well as to retrospective longitudinal cognitive decline [54].

The generalizability of these results is subject to important limitations, however. Firstly, all observations were based on cross-sectional data used to describe a longitudinal process. Secondly, the methods used to define the thresholds for tau positivity varied among the studies, and were data-driven, based on a limited number of control individuals from the same study: thus, threshold calculations require validation in separate cohorts. Thirdly, the low spatial resolution of PET and the off-target binding of [^18^F]AV-1451 could limit accurate staging of the hippocampal formation. Lastly, the classification of a large number of symptomatic individuals with a positive Aβ PET scan to Braak stage 0 [62] raises questions about either the accuracy of clinical assessment or the in vivo translation of the neuropathological staging scheme.

### Relation of tau PET to other biomarkers and apolipoprotein E

The time course of tau aggregation and its dynamic relationship to other pathophysiological features in the various tauopathies remain unclear. The theoretical models of disease progression including tau pathology were so far restricted to CSF-based measures of tau [103]. With the availability of tau PET tracers, several studies have started to investigate the relationship between regional tracer retention and other biomarkers in their population sample. Because several studies have combined CN subjects with patients in their analyses, sometimes limiting interpretation of the results, we have limited this discussion mainly to findings for patients alone or CN subjects alone.

#### Amyloid PET

**Fig. 5:**
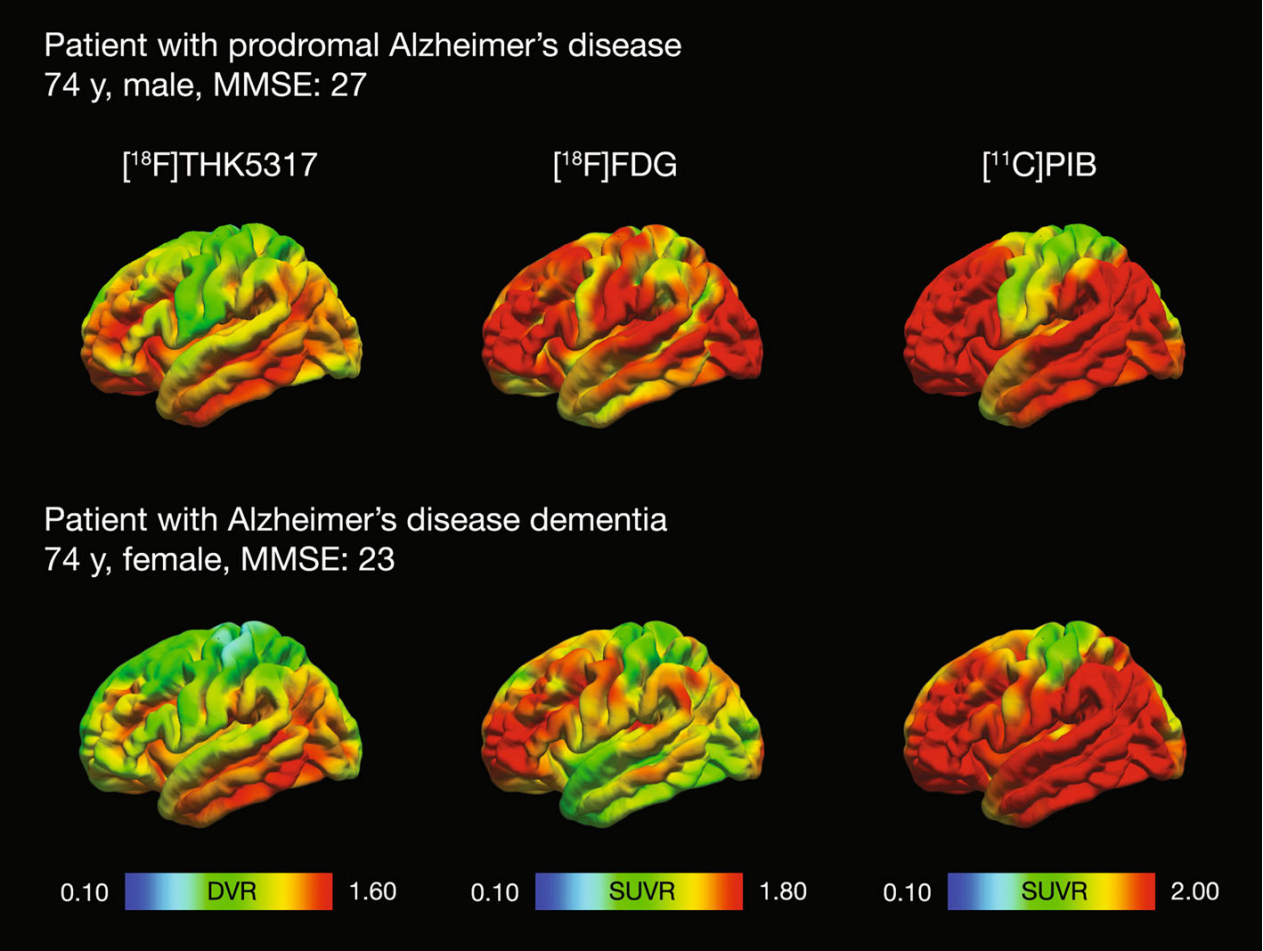
In vivo imaging of AD biomarkers in a patient with prodromal AD and in a patient with AD dementia. The retention of [^18^F]THK5317 and [^11^C]PIB are expressed with reference to the retention in the grey matter of the cerebellum; [^18^F]FDG uptake is expressed with reference to uptake in the pons. AD = Alzheimer’s disease; DVR = distribution volume ratio; FDG = fluorodeoxyglucose; PIB = Pittsburgh compound B; SUVR = standardized uptake value ratio

Studies in AD dementia patients have shown differences in the topographical retention of tau and Aβ tracers: in contrast to [^11^C]PIB, which had widespread cortical retention, the retention of THK tracers was more focal, predominantly within the temporal lobe (Fig. 5) [53, 104]. Other studies involving atypical variants of AD have also contrasted the focal cortical retention observed with [^18^F]AV-1451 to the more widespread and diffuse retention of [^11^C]PIB [61, 73, 74]. Other than these topographical differences, while no association was found between the cortical retention of [^18^F]THK5117 and [^11^C]PIB in AD dementia patients [104], positive correlations have been reported between local [^18^F]THK5317 and [^11^C]PIB retention in prodromal AD and AD dementia patients [53], as well as between temporal [^18^F]AV-1451 and global cortical [^11^C]PIB retentions in patients with MCI or AD dementia [59], suggesting the possible temporal proximity of the build-up of these two pathological processes.

#### [^18^F]FDG PET

Initial studies have reported a close correspondence between the selective retention pattern of [^18^F]AV-1451 PET and the pattern of hypometabolism with [^18^F]FDG PET in case series of patients with variants of AD (Fig. 5) [61, 73, 74]. Group level analysis in prodromal AD and AD dementia patients has revealed similar findings [105]; [^18^F]FDG uptake and [^18^F]THK5317 retention appear to be negatively correlated, primarily in frontal areas [53]. Interestingly, exploratory work on the same sample has suggested that [^18^F]FDG might play a mediating role in the association between tau pathology and cognitive decline in AD [77]. In addition, one study using [^18^F]AV-1451, [^18^F]FDG and [^11^C]PIB PET in AD patients suggested an interactive downstream effect of regional tau and Aβ on metabolism in the parietal lobe [105]. The small sample size, however, precludes any strong conclusions.

#### Structural measures

The in vivo relationship between [^18^F]AV-1451 retention and grey matter intensity measured by structural magnetic resonance imaging (MRI) was analyzed in a sample of CN subjects. A negative correlation was found, using both local (medial temporal lobe, cingulate) and distributed (widespread cortical regions) approaches [63]. Interestingly, a study by Wang et al. [64] reported that Aβ status appeared to affect the association between [^18^F]AV-1451 retention and cerebral volume; only Aβ-positive participants (CN subjects and AD patients) showed a significant association between tau imaging and volume loss. This suggests that the relationship between tau deposition and neuronal loss will only be observed in a pathological context, and highlights the importance of discriminating between CN subjects who are likely to be at a preclinical stage of AD, and those who are not.

#### Cerebrospinal fluid measures

Until recently, the only way to obtain information on tau protein in vivo was to use CSF sampling. A large number of studies have investigated the progression of CSF tau biomarkers in AD, showing a relationship between tau levels and the rate of cognitive decline [106]. Longitudinal studies on autosomal dominant AD reported that elevated CSF tau could be measured decades before the onset of symptoms [107]. The same research group also reported an unexpected finding that CSF tau level declined slightly at symptomatic stages in their studied population. Because of discrepancies reported in Aβ measurement between CSF sampling and PET imaging [108], a comparison of CSF tau levels with the newly developed tau tracers is thus of great interest. In a study of CN subjects only, significant associations were found between both CSF total and phosphorylated tau and [^18^F]AV-1451 retention in the temporal cortex [109]. Retention in other regions was associated with phosphorylated tau only. Conversely, another study reported no significant associations between either total or phosphorylated CSF tau and [^18^F]AV-1451 retention in the inferior temporal lobe in CN subjects [58]. However, significant positive associations were found when AD dementia patients were included in the analyses (in combination with CN subjects) [55, 58]. This calls for future, larger studies in patients. Of note, Chhatwal et al. [109] also reported that lower CSF Aβ_42_ in their CN subjects correlated with higher [^18^F]AV-1451 retention in neocortical but not limbic regions of the temporal lobe.

#### Apolipoprotein E

The apolipoprotein E (ApoE) ε4 allele is a well-known risk factor for developing AD. Preliminary findings on the relationship between the retention of tau tracers and ApoE ε4 status are rather discordant. In one study of AD patients, ApoE ε4 carriage was associated with [^18^F]AV-1451 retention in temporal and parietal areas, after controlling for global Aβ levels [61]. While this is consistent with findings from Cho et al. [57], which showed that the frequency of the ApoE ε4 allele was associated with higher [^18^F]AV-1451 retention in medial temporal regions in MCI and AD dementia patients, another study of MCI and AD dementia patients did not find an association between ApoE ε4 carrier status and [^18^F]AV-1451 retention [59].

Assessment of tau deposition in vivo in multimodal paradigm has raised great expectations for the understanding of the role of tau with relation to other pathological features. While preliminary, these promising studies indicate the first steps toward that goal and lay the groundwork for the testing of additional hypotheses relating to how the combination of tau imaging with other existing biomarkers may help increase diagnostic accuracy. One apparent limitation of these studies investigating the relationship between tau PET and other biomarkers, however, is that they are based on cross-sectional data, which limits interpretation of how these relationships may evolve over time.

### *Post-mortem*/*ante-mortem* comparison in humans

To date, five studies comparing *ante-mortem* tau tracer binding results with *post-mortem* findings have been performed in humans. All used [^18^F]AV-1451 in patients with non-AD pathology [97, 110–113]. In their study, Marquié et al. [111] compared the regional *in vivo ante-mortem* binding of [^18^F]AV-1451, its *post-mortem* binding on autoradiography, and tau immunostaining in three cases with 4R tau aggregates: two patients with pathologically confirmed PSP, and one patient with a MAPT mutation (P301L) presenting with an unusual histopathological phenotype of abundant cortical and white matter small grain-like tau inclusions instead of the expected NFTs and neuritic processes. The authors reported no detectable binding of [^18^F]AV-1451 to tau inclusions in these three cases, however, and no significant correlations between in vivo and in vitro binding, despite in vivo signal in basal ganglia, midbrain, and some cortical regions. A similar observation was made by Smith et al. [113] in a case of PSP, where they found that the density of tau pathology on immunostaining correlated with in vivo metabolism (measured with [^18^F]FDG PET) but not with in vivo [^18^F]AV-1451 binding. These findings suggest that the signal observed in vivo is more likely due to the presence of age-related tangles and off-target binding than to specific binding of the tracer to 4R tau aggregates. Of note, these observations regarding the MAPT mutation carrier also confirm the in vivo micro-PET findings mentioned earlier in the APPSWE-Tau transgenic mice model (carrying the human P301L tau mutation) [18].

Another case study on a MAPT mutation carrier did show good agreement between *ante-mortem* binding of [^18^F]AV-1451 in PET and *post-mortem* tau immunohistochemistry results [97]. This case, however, carried a different MAPT mutation (R406W) with 3R + 4R tangles and neurites, rather similar to AD pathology. The region with the highest [^18^F]AV-1451 retention in vivo in this case was the putamen. This region also exhibited dense tau pathology on *post-mortem* assessment, but less than in other regions such as the inferior temporal lobe, which reinforces the hypothesis of non-specific in vivo tracer binding in this region [97].

The two last studies comparing *ante-mortem* and *post-mortem* findings relied on single cases with a confirmed diagnosis of CBD (4R tau deposits) [110, 112]. Both studies reported a correlation between regional in vivo binding of [^18^F]AV-1451 and *post-mortem* tau immunostaining. However, Josephs et al. [110] reported minimal displaceable binding of [^18^F]AV-1451 on autoradiography in areas with dense 4R tau deposition (in agreement with other studies [31]), which contrasted with their in vivo observations in the same case. This again calls into question the binding properties of the tracer with respect to 4R tau pathology.

It appears, overall, that [^18^F]AV-1451 may not have enough affinity and therefore may be of limited utility for in vivo detection of tau aggregates in non-AD tauopathies. Further investigations in larger samples are required to confirm these findings.

## Future directions

This review summarizes the recent literature on the currently most promising families of tracers for specifically targeting tau in vivo. Both preclinical and early in vivo PET findings are encouraging, showing good specificity for tau and regional distribution that matches the expected pattern of tau pathology. Further work, however, is required in order to fully explain the binding properties of the tau PET tracers, and eventually to better comprehend the role of tau deposition in vivo in the pathophysiology of AD and other non-AD tauopathies.

### Further in vitro characterization is needed

The variety and complexity of tau deposits in the various tauopathies requires a great effort of characterization of tau tracers. Evidence for the specific isoforms and structural conformations of tau to which the tracers bind remains scarce. Important studies have provided preliminary but crucial information about the in vitro binding of AV-1451 in different tauopathies, reporting more extensive binding in AD brain tissue than in tissue from other diseases such as CBD, PSP, or Pick’s disease [31, 37]. These studies have also suggested that AV-1451 would not bind – or would bind to a limited extent only – to 4R tau deposits. Similar in vitro investigations are so far lacking for the other tau tracers. This matter requires further work in the near future so that a similar level of characterization is reached for all promising tracers with respect to their binding properties.

Similarly, we need to identify the binding sites of the tracers, along with the number of sites to which they bind. Studies have suggested multiple binding sites on tau for THK tracers, and already comparisons between tracers suggest that some target the same sites (AV-1451 and MK-6240) [23] while others do not (AV-1451 and PBB3) [33]. Future studies investigating the location and accessibility of the binding sites in different types of tau deposits will add information essential to our understanding of the tracers distribution patterns. Indeed, it is likely that the accessibility of a given tracer to its binding site(s) will be affected by the isoform and conformation of tau in the targeted deposit.

### Different tracers for different diseases?

Because the different tracers come from distinct chemical families, they are likely to bind differently to tau deposits. While all tracers described in this review have good affinity to tangles and have shown a similar binding pattern in vitro and in vivo in the AD brain, which also resembles the pathological pattern described with immunostaining, discrepancies seem to exist between the tracers in their binding to tau deposits in non-AD cases. There is, however, very little in vitro evidence with regard to the binding of tau tracers in non-AD tauopathies, especially for THK tracers and PBB3, and, to date, no head-to-head comparisons between tracers (in the same patient populations), either in vitro or in vivo. This represents an important future challenge, as such results would provide much required understanding of tracers specificity, and would help determine whether some tracers may be more suitable for tracking tau deposition in some tauopathies than in others. In addition, new tracers currently under development or undergoing preclinical assessment may have advantages over those already described, such as lower off-target binding. Comparison between these candidates and other tau tracers will also be required.

### Off-target binding

The off-target binding observed for all the tau tracers, both in vivo and in vitro, is a major issue. There is currently a great effort to try to define what this off-target may represent. The fact that some of the regions showing off-target binding in vivo are regions where specific binding would be expected in some tauopathies is of particular concern; this is the case, for instance, for the basal ganglia in CBD and PSP. Early evidence from in vitro work on AV-1451 has suggested that the signal observed in several brain regions could be due to binding to different features, such as pigmented and mineralized structures [31], MAO-A [23] but also MAO-B [114, 115]. Further work to resolve the question of this off-target binding is thus required.

### The use of tau animal models

Transgenic mouse models of tau deposition offer the potential to assess the ability of tau tracers to track the temporal and regional deposition of tau. Based on the few in vivo micro-PET studies thus far performed, there is evidence that certain mouse models may not be suitable for investigating the binding of at least some of the tau tracers. Additional studies using various mouse models to assess in vivo binding will be of great interest, and are crucially needed for the future development and testing of novel anti-tau therapies.

### Assessment of tau propagation in vivo

In parallel with in vitro characterization studies, the large body of work that became rapidly available on in vivo retention of tau tracers in humans has provided us with important insights into tau deposition. Studies have attempted to stage tau progression in vivo in AD patients by classifying individuals into PET-based Braak stages according to the retention pattern of tau PET tracer. Future studies using a longitudinal design as well as pathological confirmation will be necessary for validation of these in vivo staging models. In addition, tau PET imaging will likely prove of use in clarifying the role of tau pathology with respect to other AD biomarkers [103] and in the operationalization of novel classification schemes [116]. Increasing evidence from in vivo studies suggests that, while they do not share the same deposition patterns, PET-measured tau and amyloid deposition in AD are associated in various areas of the brain. This fits with the hypothesis of a dynamic interaction between tau and Aβ pathology. Again, the absence of longitudinal studies precludes us from drawing conclusions, but these findings already illustrate the potential of in vivo investigations to further our understanding the dynamic process of tau deposition and its interaction with other key actors in the disease. The emergence of longitudinal multimodal data in a near future should allow to test how the combination of tau imaging with other existing biomarkers may help increase diagnostic accuracy.

As mentioned above, there has been great interest in recent decades in studying familial forms of AD, as these enable the investigation of biological mechanisms occurring in the disease course even before the onset of clinical symptoms. One case report [117] described a high in vivo retention of [^18^F]AV-1451 in a symptomatic carrier of a presenilin-1 mutation (Thr116Asn) [118]. While very little is known about this particular mutation and its underlying pathology, which precludes further conclusions, this result does offer potential for more investigations in familial AD, particularly future longitudinal assessments in patients in the presymptomatic stages; this would certainly allow the collection of valuable information on the role of tau in the disease course at the earliest stages.

The opportunity of assessing the pathophysiological role of tau in vivo in tauopathies other than AD has stirred great interest, and promising findings have emerged. While most come from case studies, one study on groups of patients illustrated for the first time the potential of regional measurement of [^18^F]AV-1451 retention to discriminate between two tau-related diseases (dementia with Lewy bodies and AD) [94]. Further replication studies in larger sample sizes are however needed. In vivo work in other diseases affected by tau, such as chronic traumatic encephalopathy, is also of interest, and preliminary reports have started to emerge [119, 120]. Assessment of the specificity of the tracers in other proteinopathies, such as TDP-43-related diseases, will also be of interest.

Because of the novelty of tau radiotracers, most of the work comparing their intra-individual *ante-mortem* and *post-mortem* binding has to date used animal models. Among the few reports on humans, discrepancies have been reported between the in vivo and in vitro binding of AV-1451 in cases with 4R tau deposits [110], highlighting the important questions of how different forms of tau may affect tracer binding, and how transposable in vitro observations are to in vivo PET. It is possible that dynamic processes occurring in vivo, which are not possible to assess in *post-mortem* tissue, may play a role. Future studies in pathologically confirmed cases investigating the correspondence between results from in vivo tau PET and *post-mortem* tau staining are imperative in order to shed more light on this matter.

In addition, there remain methodological concerns regarding quantification in the assessment of in vivo tau tracer retention in non-AD tauopathies. Specifically, while reference-tissue models have been shown to describe retention well over time for most tracers, reference region selection could be an issue, as the commonly used cerebellum can be affected by tau pathology in some non-AD tauopathies as well as in the later stages of AD [53]. This matter deserves more attention, especially when larger cohorts are studied. Harmonization of quantification methods across studies and possibly across tracers (as it is now happening for Aβ PET with centiloid scaling [121]), will also be required for better comparison of findings.

### Tau PET versus tau CSF

Some studies, but not all, have reported an association between regional tau PET retention and CSF tau levels. Although exploratory, these findings seem to indicate that, as with Aβ biomarkers, CSF and PET-based measures of tau may result from the same pathological processes, but may not invariably mirror one another, instead providing complementary information. These investigations will need to be pursued in larger AD cohorts, and possibly with new CSF assays.

### The use of tau PET in clinical trials

As a pathological hallmark in AD and other tauopathies, tau aggregates have been an attractive target for immunization therapy. In the absence of efficient treatments able to stop disease progression, and with the failure of several therapies aiming to reduce Aβ load, clinical trials focusing on the inhibition of tau aggregation have emerged [122]. While few results from such trials are as yet available, a recent phase III study testing the tau protein aggregation inhibitor Methylthioninium reported no benefit from the treatment in patients with mild to moderate AD [123]. Further results are however expected from ongoing phase II and III trials after promising early results [124], which will hopefully report positive effects of treatments. Along with this increasing interest to develop novel anti-tau therapies, there is a compelling need to incorporate tau PET imaging as a reliable outcome measure to evaluate drug efficacy. Therefore, as the field of tau PET imaging advances, tau PET will become important to evaluate the therapeutic effects of such drugs on tau burden in the brain, and will certainly be increasingly incorporated in future clinical trials.

## Conclusions

In conclusion, the growing number of studies investigating tau PET has provided exciting and encouraging results on the usefulness of tau PET tracers in exploring tau pathology in various diseases. New paths are now becoming open to us, and more in-depth work is required to further our understanding of the role of tau in AD and other tauopathies.

## Abbreviations

AD: Alzheimer’s disease
ApoE: Apolipoprotein E
Aβ: Amyloid-beta
CBD: Corticobasal degeneration
CN: Cognitively normal
CSF: Cerebrospinal fluid
FDG: Fluorodeoxyglucose
MAO: Monoamine oxidase
MAPT: Microtubule-associated protein tau
MCI: Mild cognitive impairment
MRI: Magnetic resonance imaging
NFTs: Neurofibrillary tangles
PART: Primary age-related tauopathy
PET: Positron emission tomography
PHFs: Paired-helical filaments
PIB: Pittsburgh compound B
PSP: Progressive supranuclear palsy
ROI: Region of interest
SNAP: Suspected non-AD pathophysiology
SUVR: Standard uptake value ratio
